# Tet-Transgenic Rodents: a comprehensive, up-to date database

**DOI:** 10.1007/s11248-012-9660-9

**Published:** 2012-11-23

**Authors:** Kai Schönig, Sabine Freundlieb, Manfred Gossen

**Affiliations:** 1 Central Institute of Mental Health, Medical Faculty Mannheim / Heidelberg University, Mannheim, Germany; 2 TET Systems GmbH, Heidelberg, Germany; 3 Berlin-Brandenburg Center for Regenerative Therapies (BCRT), Föhrer Str. 15, 13353 Berlin, Germany; 4 Max Delbrück Center for Molecular Medicine, Berlin, Germany

**Keywords:** Tet system, Transactivator lines, Responder lines, Database

## Abstract

Here we introduce the “Tet-Transgenic Rodents” database, documenting most of the published Tet-transgenic mouse lines generated in the past 2 decades. Aside from the >500 mouse lines listed, it also includes the first of the recently reported Tet-transgenic rat models. Since the Tet technology comprises two essential components, a *cis*-acting promoter (P_tet_) and a *trans*-acting transactivator, the database has been organized accordingly. One section of the database summarizes the different transgenic mouse lines carrying mostly tissue specific promoters driving the Tet transactivator. Another section covers transgenic mouse lines carrying responder transgenes under P_tet_ control. The few existing rat transgenic lines are listed correspondingly. It is the purpose of this database to facilitate the repeated use of preexisting, validated transgenic lines as a shortcut for further research.

## Background

The development of transgenic mouse technologies has made tremendous contributions to our in-depth understanding of developmental and physiological processes in mammalian organisms. Moreover, transgenic disease models provide valuable information on pathophysiological processes, e.g. in cancer or in neurological and metabolic disorders. The sophistication by which customized mouse models can be generated has been aided in particular by two technologies based on functional elements derived from prokaryotes: the Cre/loxP system and the Tet system (Lewandoski 2001). They convey different forms of conditionality to transgene expression. Generally speaking, Cre/loxP allows for irreversible, stable modification of the mouse genome via Cre recombinase action. In contrast, the Tet system facilitates the reversible modification of the transcriptome, due to the action of tetracycline-controlled transcription factors.

The awareness of the scientific community to the numerous Cre expressing and loxP site modified transgenic mouse lines was considerably raised through databases maintained by researchers in this field (Nagy et al. 2009; see also http://creline.org for a compilation of the various databases available). In contrast, the large number of transgenic mice generated with components of the Tet regulatory system has so far not been adequately compiled.

With this letter we want to convey a sense of the multitude and diversity of such existing animals by briefly introducing a database documenting the majority of published Tet-transgenic mouse lines. We hope that this freely available and continuously updated collection will foster the collaboration between researchers intending to create new Tet-transgenic rodent models.

## The Tet system

Over the past 2 decades tetracycline-controlled gene expression established itself as the foremost principle for small molecule-inducible gene expression in mammalian cells. Both the Tet-Off (Gossen and Bujard 1992) and Tet-On (Gossen et al. 1995) system also found widespread application in transgenic mice. These binary regulatory systems require (1) a transactivator transgene, based on a fusion between the prokaryotic Tet repressor (TetR) and a eukaryotic transactivation domain and (2) a synthetic promoter (P_tet_) designed to be transcriptionally silent unless bound by the transactivator. This is achieved by integration of TetR binding sites (the tet operators, *tet*O) proximal to the core promoter, rendering gene expression of the regulatory system responsive to doxycycline. This tetracycline derivative binds to the TetR moiety of the transactivators, thereby controlling their DNA binding either in a positive (Tet-On) or negative (Tet-Off) way. In the vast majority of applications of the Tet system in transgenic mice, transactivator lines and responder lines (with the gene of interest under P_tet_ control) have initially been generated independently. Double transgenic mice are then obtained by breeding. This strategy has several advantages:

1. Expression units for the transactivator and responder transgene do not interfere with each other’s activity, as it has been observed for co-integrated transgenes.
2. Both the responder and the activator line can be characterized independently of each other (e.g. using established reporter lines), with optimal lines chosen for subsequent crosses and, most importantly in the context of this letter,
3. Individual lines generated with a substantial effort can be “re-used” for subsequent experiments. For example, several “popular” transactivator mouse lines like CCSP-rtTA (Tichelaar et al. 2000) or Keratin14-rtTA (Nguyen et al. 2006) have been re-used in numerous subsequent studies by other laboratories to study questions in lung and skin biology, respectively. Likewise, specific responder lines e.g. coding for a tightly controlled Diphtheria toxin (Lee et al. 1998) have been used in a variety of scientific questions to ablate cells of many different tissues and organs like cardiac muscle, liver, pancreas, brain etc., depending on the transactivator expression pattern. Thus similar or analogous questions as in the original study may be asked, and results can be directly compared as the animals are isogenic for either the transactivator or the responder transgene. Alternatively, researchers often take advantage of well characterized transgenic lines e.g. expressing a transactivator in a defined organ or tissue, which then can be bred to entirely different responder transgenes, thus addressing a multitude of unrelated questions. For more examples for these scenarios, see Schönig et al. (2010).

## The database

Maximizing the potential benefits of utilizing previously established Tet-transgenic lines requires knowledge of their existence. By collecting such information and also by highlighting the availability of numerous of these Tet mouse lines in transgenic mouse repositories we wish to contribute to further dissemination of the technology and to ease access to specific lines. Lastly, we also started to collect information on Tet-transgenic rats, the number of which presumably will increase rapidly within the next years.

**Fig. 1:**
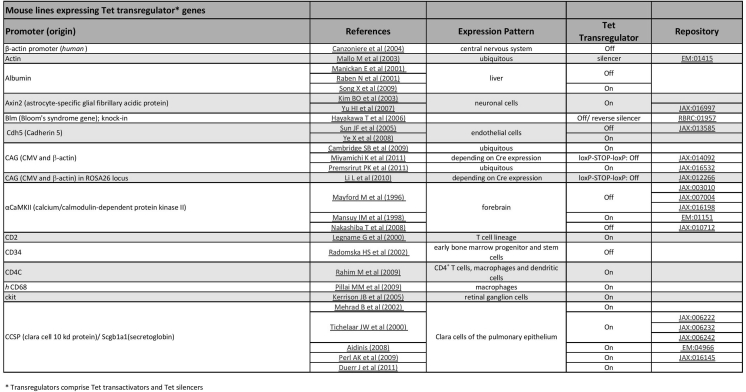
Screenshot of the table for mouse lines expressing Tet transregulator genes. Animals are ordered alphabetically according to the promoter controlling transregulator expression. The Pubmed links given refer to the original publication of the transgenic line, not to subsequent “recycling” experiments. For example, we are aware of 3 independently generated mouse lines in which the albumin promoter drives expression of Tet transregulators, with 2 lines utilizing a Tet-Off type and 1 line utilizing a Tet-On type transactivator. The expected expression pattern in cell types and/or organs are provided. Where applicable, repositories distributing specific animals are indicated, including an online link. Repositories covered are: The Jackson Laboratories (http://jaxmice.jax.org/research/tet.html). MMRRC (http://www.mmrrc.org/catalog/StrainCatalogSearchForm.php?search_query=tTA%2C+rtTA%2C+tetO). EMMA (http://www.emmanet.org/mutant_types.php?keyword=tet_expression_system). RIKEN BRC (http://www.brc.riken.jp/lab/animal/catalogue/Tetsystem.html)

**Fig. 2:**
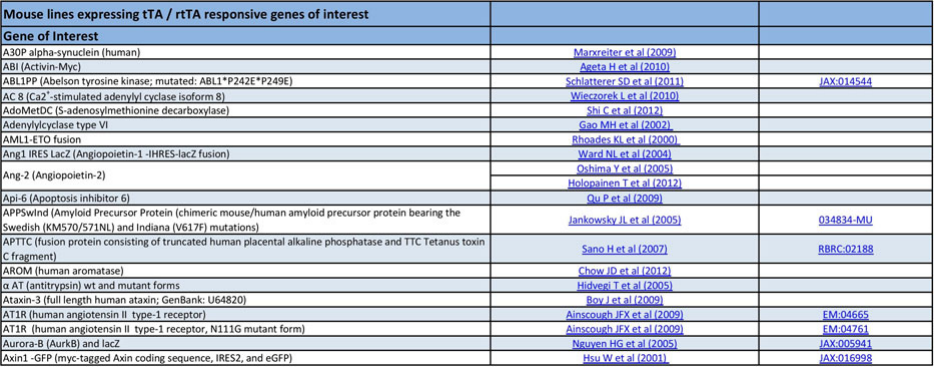
Screenshot of the table for mouse lines expressing tTA/rtTA responsive genes of interest. Animals are ordered alphabetically according to the gene placed under control of P_tet_. For information regarding the references and repositories, refer to Figure 1

**Fig. 3:**
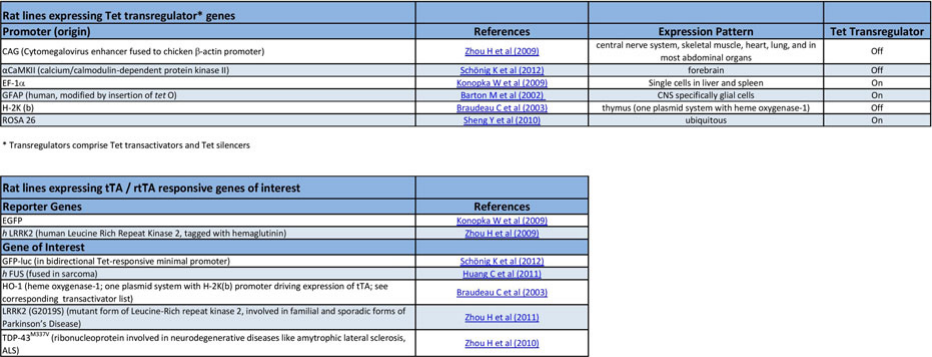
Screenshot of the combined table for rat lines expressing either Tet transregulators or tet responsive genes. The tables for Tet transregulator and Tet-responsive transgenic rats are structured according to the respective tables for Tet-transgenic mice

Figures 1, 2, and 3 show screenshots of exemplary sections of the database as it will appear when accessed online. The figure legends provide a short introduction to the database including links to repositories distributing many of the transgenic animals. The database itself can be found at http://www.tetsystems.com/fileadmin/tettransgenicrodents.pdf.

Finally, we like to apologize to those colleagues who published novel Tet-transgenic lines and whose work escaped our attention. We like to encourage you to contact us via info@tetsystems.com to have your original transactivator or responder line incorporated into the database.
